# Beyond multiculturalism? Rethinking Japan's “tabunka-kyosei” through Axel Honneth's theory of recognition

**DOI:** 10.3389/fsoc.2025.1653520

**Published:** 2025-09-09

**Authors:** Hiroyuki Ishimatsu

**Affiliations:** Center for Liberal Arts, Fukuoka Institute of Technology, Fukuoka, Japan

**Keywords:** tabunka-kyōsei, multiculturalism, Axel Honneth, recognition theory, Japanese immigration policy, cultural diversity, social solidarity, institutional recognition

## Abstract

**Introduction:**

In Japan, the concept of *tabunka-kyōsei* (multicultural coexistence) has emerged in response to the growing number of foreign residents, yet its ideological background and policy implications remain insufficiently examined in international discourse. Unlike multiculturalism in Europe, Canada, and Australia, it has evolved under Japan's unique social conditions and remains conceptually distinct.

**Methods:**

This study adopts a normative theoretical approach, analyzing tabunka-kyōsei through Axel Honneth's theory of recognition. Selected local initiatives are used illustratively to explore broader conceptual implications without empirical validation of specific cases.

**Results:**

The analysis traces the development of foreign resident policies in Japan, identifies the distinctive ideological and institutional features of tabunka-kyōsei, and compares them with Western multiculturalism. Differences include limited rights-based frameworks and an emphasis on exchange and mutual understanding over institutional recognition.

**Discussion:**

Applying Honneth's three-layered framework of love, law, and solidarity, the study argues that institutional recognition and social solidarity are central to evaluating Japan's multicultural practices beyond cultural tolerance. The findings contribute to normative debates on tabunka-kyōsei and offer insights for designing inclusive policies in contemporary Japan.

## 1 Introduction

In Japan, the term *tabunka-ky*ō*sei*, meaning “multicultural coexistence” or “multicultural coliving”, is widely recognized as a vision for a future multicultural society. It developed in response to the increase in foreign residents, but its ideological background and policy implications have not been sufficiently compared with or examined in an international context (Miyajima, 2009). While multiculturalism is an established political theory and policy framework in Europe, Canada, Australia and New Zealand, Japanese tabunka-ky$\bar{\text{o}}$sei is a concept that emerged in response to Japan's unique social circumstances and, as a term, has a structure that is difficult to connect to international theoretical frameworks—even though some Japanese theorists have attempted to translate it into a recognizable word.^1^ In this paper, tabunka-ky$\bar{\text{o}}$sei is intentionally used in its original romanized Japanese form, rather than translated, in order to preserve its conceptual specificity. As a result, while Japan has accumulated practical experience in tabunka-ky$\bar{\text{o}}$sei, it has remained outside the scope of international theoretical discussions.

The purpose of this paper is to examine the characteristics, challenges, and potential of Japan's tabunka-ky$\bar{\text{o}}$sei from a political theory perspective, particularly through the lens of Axel Honneth's theory of recognition. Rather than conducting empirical validation of specific policy cases, this paper takes a normative theoretical approach, incorporating selected local initiatives illustratively to examine the broader conceptual implications. First, we will provide an overview of the historical background of the increase in foreign residents in Japan and the emergence of tabunka-ky$\bar{\text{o}}$sei as a policy response. Next, we will compare this with the ideological and institutional characteristics of “multiculturalism” in Europe and the United States, clarifying the differences and commonalities between the two (Taylor, 1994; Kymlicka, 2001; Parekh, 2000). Finally, we will evaluate the idea and policies of tabunka-ky$\bar{\text{o}}$sei using Axel Honneth's theory of recognition. Unlike the argument emphasizing the importance of belonging to cultural communities necessary for identity formation, as seen in Canadian multiculturalism (Taylor, 1994), Honneth's theory emphasizes the necessity of recognition in both the legal domain as citizens and the social domain (Honneth, 1996). This makes it meaningful for evaluating the field of tabunka-ky$\bar{\text{o}}$sei in Japan, which focuses on language (Japanese) education, cultural exchange support, and livelihood support. This paper examines Japan's tabunka-ky$\bar{\text{o}}$sei policies theoretically with reference to Honneth's three-layered structure of “love,” “law,” and “solidarity.” In particular, it questions what implications the establishment of recognition relations in the areas of institutional recognition and social solidarity, rather than merely respecting cultural differences, has for the concept of tabunka-ky$\bar{\text{o}}$sei.

While several local practices are briefly introduced, they are not the object of empirical evaluation but serve to illustrate how theoretical concepts of recognition may be observed or challenged in real-world multicultural settings. The significance of this paper lies primarily in its contribution to political theory and social philosophy. In Japan tabunka-ky$\bar{\text{o}}$sei is often discussed in terms of policy practices or operations at the local government level, but it is necessary to redefine it as an ideal and to construct a theoretical framework in order to establish a normative vision of the direction society should take. Abstract concepts provide a perspective from which concrete practices can be relativized and evaluated, while theory gives meaning and direction to trial and error in the field. The difference between the perspective of those in the field and that of observers can be compared to the roles of a soccer player and a coach.

In addition, there is the significance of applying theory to the reality of multicultural societies. For example, in Canada, multiculturalism in practice and political theory have reinforced each other to shape institutions (Kymlicka, 2002). In Japan as well, as efforts spanning diverse areas such as employment support, living support, disaster education, and language policies are advancing in foreign resident communities (e.g., Hamamatsu City and $\bar{\text{O}}$izumi Town), it is meaningful to structure these practices theoretically, to confer institutional legitimacy upon them, and simultaneously to provide a perspective for identifying future challenges (Miyajima, 2009).

## 2 Materials and methods

This study combines theoretical analysis grounded in political philosophy with illustrative field observations drawn from local multicultural contexts in Japan. The theoretical framework was constructed through an in-depth review of key literature on multiculturalism and recognition theory, including works by (Taylor 1994), (Kymlicka 2001), (Parekh 2000), and (Honneth 1996). These texts provided the conceptual tools necessary to evaluate Japan's tabunka-ky$\bar{\text{o}}$sei in light of internationally recognized normative frameworks. In parallel, field visits and semi-structured interviews were conducted between April and May 2025 in regions with a high proportion of foreign residents. Specifically, the author visited Hamamatsu City^2^ (Hamamatsu International Association, 22 April 2025), $\bar{\text{O}}$izumi Town^3^ in Gunma Prefecture ($\bar{\text{O}}$izumi Town Hall and $\bar{\text{O}}$izumi International Association, 23 April 2025), and the Multicultural Center Tokyo (2025)^4^ (a private NPO-run free school for foreign children, 24 April 2025). Additionally, interviews were held with staff from the Saga Prefectural Multucultural Promotion Division and SPIRA^5^ (Saga Prefecture International Exchange Association, 19 May 2025). These interviews were conducted solely with adult professional stakeholders and were anonymized. While they provide contextual insights, the interviews are not treated as primary empirical data in a social scientific sense, and no formal coding or triangulation procedures were applied. The integration of theoretical reflection and empirical observation enables a multi-scalar analysis: connecting abstract normative ideals with the lived realities of tabunka-ky$\bar{\text{o}}$sei in practice. This methodology helps identify both the conceptual strengths and the limitations of Japan's current approach to tabunka-ky$\bar{\text{o}}$sei.

## 3 Background, philosophy, and policies of tabunka-ky$\bar{\text{o}}$sei in Japan

### 3.1 Japan's multicultural situation and recent trends

Japanese society has long held the self-image of a “single-ethnic nation” based on cultural homogeneity (Oguma, 2002), but in reality, people from diverse cultural backgrounds coexist. The Ainu, as an indigenous people, have preserved their unique language, religion, and way of life centered in Hokkaido. However, they faced social exclusion due to assimilation policies implemented after the Meiji period. In 2008, they were officially recognized as an indigenous people by the National Diet, and the Ainu Policy Promotion Act was enacted in 2019. Nevertheless, disparities in education, employment, and living conditions remain unresolved (Cabinet Secretariat, 2019).

Zainichi Koreans, that is, Koreans and their descendants who came to Japan before or during World War II and settled there, have faced issues of legal status and discrimination while establishing roots in Japanese society as the so-called “old-comers” (Miyajima, 2004). Many of them lost their nationality after the war and were granted the institutional status of “special permanent residents (tokubetsu eij$\bar{\text{u}}$sha),” but their marginal position in Japanese society has persisted for a long time. As of the end of 2023, there were ~280,000 Korean residents in Japan (Immigration Services Agency of Japan, 2024).

The “new-comer” population that arrived after the 1990s includes South Americans, primarily Japanese Brazilians, as well as immigrants from the Philippines, China, Vietnam, and other countries. In particular, the 1990 amendment to the *Immigration Control Act* granted Japanese Brazilians the right to engage in unskilled labor, leading to the settlement of many South Americans in industrial areas (Tsuda, 2003). In $\bar{\text{O}}$izumi Town, Gunma Prefecture, approximately 20% of the population are foreign nationals, with over half being Brazilians (Town of Ōizumi, 2023). In recent years, young workers from Vietnam, Myanmar, and Indonesia have increased through the Technical Intern Training Program,^6^ with approximately 360,000 technical interns in 2023, about 50% of whom are Vietnamese (Immigration Services Agency of Japan, 2024).

This increase in foreign residents is closely related to Japan's severe population decline and aging society. According to the Ministry of Internal Affairs and Communications' population estimates (January 2024), Japan's total population was approximately 124.08 million, marking 13 consecutive years of decline. The elderly aged 65 and over accounted for 29.1% of the population, setting a new record high. The working-age population aged 15 to 64 has declined to 59.4%, and labor shortages are becoming particularly severe in sectors such as construction, nursing care, and food services (Statistics Bureau of Japan, 2024).

Against this backdrop, the “Specific Skills System” (Tokutei-gin$\bar{\text{o}}$ seido) was introduced in 2019. This system allows foreign nationals with certain skill levels and Japanese language proficiency to work in 14 specific industries (including healthcare, construction, agriculture, accommodation, and food services), with the “Specific Skills 1” (Tokutei-gin$\bar{\text{o}}$ 1) residence status allowing for a maximum stay of 5 years. Additionally, those with advanced skills can obtain “Specific Skills 2” (Tokutei-gin$\bar{\text{o}}$ 2) status, which allows them to bring their families and extend their stay. As of April 2024, approximately 180,000 people were residing in Japan under the Specific Skills System, with further increases expected (Immigration Services Agency of Japan, 2024).

### 3.2 The concept and policies of tabunka-ky$\bar{\text{o}}$sei

#### 3.2.1 The origin and definition of tabunka-ky$\bar{\text{o}}$sei policies

In 2006, the Ministry of Internal Affairs and Communications announced the *Plan for the Promotion of Tabunka-ky*ō*sei in Local Communities* which marked the institutional launch of Japan's tabunka-ky$\bar{\text{o}}$sei policies (Ministry of Internal Affairs and Communications (MIC), 2006).^7^ The plan defines tabunka-ky$\bar{\text{o}}$sei as “the coexistence of people of different nationalities, ethnicities, and other backgrounds who recognize each other's cultural differences and build equal relationships while living together as members of the local community” (Ministry of Internal Affairs and Communications (MIC), 2006). This plan is based on the principle of “respect for cultural differences,” but it does not necessarily involve the protection of rights or institutional recognition of cultural groups that figure in Western multiculturalism (CLAIR, 2020).^8^^,^^9^

#### 3.2.2 Policy implementation structure and the role of local governments

The Ministry of Internal Affairs and Communications is responsible for formulating policy guidelines and sharing best practices, while the implementation of policies is delegated to local governments. According to the latest 2024 survey by the Ministry of Internal Affairs and Communications, 995 local governments, approximately 56% of all 1,741 municipalities in Japan, have established Tabunka-ky$\bar{\text{o}}$sei Promotion Plans (Ministry of Internal Affairs and Communications, 2024). In areas with a high proportion of foreign residents, unique initiatives are being developed. For example, Hamamatsu City in Shizuoka Prefecture issued a *Hamamatsu Intercultural City Vision* in 2001 and established a comprehensive support system that includes support for schools for foreign residents, the provision of medical interpreters, and the operation of an international center through citizen collaboration (Hamamatsu City, 2023). The ratio of foreign residents in the city has reached approximately 4.3%. In Iwate Prefecture, located in northeastern Japan, disaster multilingual support systems were strengthened in the wake of the Great East Japan Earthquake, and efforts are being made to promote disaster prevention education for foreign residents and connect them with the local community (Iwate Prefectural Government, 2016).

#### 3.2.3 Consequentialist nature and planned harmonious coexistence

Japanese tabunka-ky$\bar{\text{o}}$sei is essentially consequentialist, as coexistence seems to be considered to exist when friction is avoided (Miyajima, 2009). For this reason, understanding of different cultures and regional harmony are emphasized, while cultural differences and rights issues tend to be overlooked. For example, while Japanese language acquisition is taken for granted as a *prerequisite for daily life*, the preservation of one's mother tongue and the establishment of institutional interpretation services are treated as supplementary measures (Tsuda, 2003). This harmonious view of coexistence (ky$\bar{\text{o}}$sei) tends to create pressure for assimilation into the majority culture. As a result, a structure is preserved in which minorities are allowed to participate in *cohesion* only by conforming to the norms of the majority.

#### 3.2.4 Theoretical and practical issues

Theoretically, the problem lies in the fact that the concept of tabunka-ky$\bar{\text{o}}$sei is constructed from the perspective of the majority (Ishihara, 2004). As pointed out by feminist criticism, it is important to note that *inclusion* and *coexistence* can often function as strategies of domination (Fraser, 2000). Minorities are only made visible when they are deemed harmless by the majority population.

In practice, discretionary responses by local governments are the mainstay, and discussions on institutional legitimacy and rights protection remain underdeveloped (Arudou, 2015). While cultural exchange events and mutual understanding programs are widely conducted, fundamental discussions on how to define the *units of recognition* or *boundaries of rights* for foreigners and minority groups tend to be avoided.

In particular, as long as foreign residents remain a minority, problems do not surface, but once their population exceeds a certain threshold and cultural symbols such as religious facilities and signs in their native languages become visible, friction among residents and the limitations of administrative responses become apparent. Until now, tabunka-ky$\bar{\text{o}}$sei in Japan has prioritized on-the-ground coordination and conflict avoidance over institutional recognition of culture and collective rights (Ministry of Internal Affairs and Communications, 2022).

While the social pressure to conform (d$\bar{\text{o}}$ch$\bar{\text{o}}$-atsuryoku) is often recognized and self-reflected upon by Japanese citizens themselves, its direct linkage to Confucianism remains a matter of scholarly debate. In my view, although values associated with Confucianism—such as hierarchy, filial piety, and respect for harmony—may overlap with social norms in Japan, it is difficult to establish a clear causal relationship between Confucian philosophy and Japan's current approach to cultural diversity. Furthermore, if one were to assert such a relationship, comparative studies with countries such as South Korea, where Confucian influence is arguably stronger, would be necessary. Nevertheless, some scholars have argued that Confucian legacies in East Asia may contribute to societal norms that prioritize group harmony and hierarchies, potentially shaping the assimilation-oriented tendencies seen in Japanese multicultural policies.^10^

### 3.3 Examples of current challenges to tabunka-ky$\bar{\text{o}}$sei in Japan

In 2018, the Japanese government adopted the Comprehensive Measures for the Acceptance and Coexistence of Foreign Human Resources (MIPEX, 2020), followed by the 2019 launch of the Specified Skilled Worker visa system, which aimed to expand low-skilled labor inflow. While OECD and IOM both position Japan as a de facto country of immigration, the Japanese government explicitly denies adopting any “immigration policy” (MIPEX, 2020). In practice, the number of foreign workers in Japan has continued to rise, reaching ~2.3 million as of October 2024 (Ministry of Health, Labour and Welfare, 2024). Although long-term settlement among foreign residents is progressing, the government still tends to view them as temporary labor. Even under the Specified Skilled Worker (SSW2) program, where limited family reunification and permanent residency pathways have been introduced, legal and procedural barriers to naturalization and permanent residence remain high.^11^ In recent years, Japanese society has been rapidly becoming more multicultural due to an increase in the number of foreign residents (Ministry of Justice, 2024). Within this context, friction and challenges are emerging in specific local communities. The conflict between Kurdish residents and local residents in Warabi City,^12^ Saitama Prefecture, is one such example. With a significant number of Kurds residing in the area, tensions have arisen between residents as cultural differences become visible in public spaces and local communities, leading to media coverage and societal attention (Mainichi Shimbun, 2023).

In addition, there are growing calls among some religious groups, such as Muslims, for burial of their dead, but the difficulty of reconciling this with Japan's cremation-centered customs is compounded by the fact that only seven cemeteries across the country accept Muslim burials (Asahi Shimbun, 2022; Kojima, 2023). In particular, local governments are increasingly required to respond to situations that were not necessarily anticipated in the institutional framework, which does not necessarily take into account such diverse views on death and the afterlife.

These cases illustrate that Japan, which has not established clear ideals or systems as an *immigrant nation*, is facing a multicultural reality. Moreover, this is not limited to a single administrative domain such as *foreign resident services*, but involves fundamental structures of society, including regional lifestyles, values, religion, rituals, and the use of public spaces.

## 4 Multiculturalism and Tabunka-ky$\bar{\text{o}}$sei

Multiculturalism policies adopted by countries such as Australia, Canada, and the United Kingdom are based on the principle that the cultural and ethnic identities shared by members of a community are essential, and that cultural diversity contributes to the common good. These policies aim to guarantee human rights universally while publicly supporting the maintenance and reproduction of collective cultural traditions. On the other hand, Japanese tabunka-ky$\bar{\text{o}}$sei recognizes the importance of cultural and ethnic identities as part of the dignity of individuals, but stops short of to publicly support their maintenance and reproduction. Here, we will clarify the characteristics of tabunka-ky$\bar{\text{o}}$sei by comparing it with the main points of representative theories of multiculturalism.

### 4.1 Theories of multiculturalism

#### 4.1.1 Charles Taylor: multiculturalism based on recognition

Canadian philosopher Charles Taylor argued in his seminal work, The Politics of Recognition, that the demand for recognition in modern society underlies political movements such as nationalism, feminism, and multiculturalism (Taylor, 1994). According to Taylor, humans are “self-interpreting animals” who form their identities in relation to others. Therefore, misrecognition is not merely a matter of disrespect, but can become a *serious wound* that leads to self-hatred (Taylor, 1994, pp. 25–26, 65–66).

Taylor's argument calls for the guarantee of universal citizenship and compensatory consideration for groups that have been culturally and historically disadvantaged. He proposes a *dual policy* framework to balance universality and differences. For example, Quebec's language protection policies and the self-determination rights of indigenous peoples are defended on this basis (Taylor, 1994, pp. 52–61). Taylor's argument demonstrates that the existence of multiple cultures is not merely a matter of the rights of their members, but also has value in terms of the diversity and richness of the public sphere itself (Taylor, 1994, pp. 58–61, 71–73). This position is consistent with Raz's argument that diverse values and cultural options must actually exist in society for *autonomous choice* to be possible (Raz, 1986).

However, such cultural preservation policies also carry the risk of excessive segregation and exclusion, and it has been pointed out that separate education, for example, may hinder the integration of society as a whole. While Taylor's theory provides a humanistic and philosophical justification for multiculturalism, there is still room for consideration regarding its policy implications.

#### 4.1.2 Will Kymlicka: multicultural citizenship and institutional justice

Another Canadian theorist Will Kymlicka develops a theory of multicultural citizenship based on the premise that cultural identity is indispensable for the realization of a good life for individuals (Kymlicka, 1998). A distinctive feature of his argument lies in his classification of minority groups according to their historical backgrounds, and in proposing institutional rights appropriate to the circumstances of each group. Specifically, Kymlicka distinguishes between multination states and polyethnic states, using the term “multination” (rather than “multinational”) to highlight the political and historical dimensions of national identity.

A multination state refers to a country that includes multiple nations within its borders, such as indigenous peoples or historically settled ethnic minorities who were often subjected to assimilationist policies. In such cases, Kymlicka argues for the necessity of external protections—institutional rights that allow these groups to preserve their cultural practices and self-governance as a form of collective autonomy.

A polyethnic state, by contrast, refers to societies that have experienced large-scale immigration, where cultural minorities have migrated voluntarily. Here, the primary concern is how to accommodate cultural diversity while facilitating integration into the mainstream society. Rather than autonomy, the focus is on multicultural rights within shared institutions.

Kymlicka further classifies the rights of minorities into two types: internal restrictions and external protections. Internal restrictions refer to limitations imposed within a group, such as preserving traditional norms or values, but Kymlicka is cautious about these, emphasizing that such restrictions should not infringe upon the individual rights and freedoms of group members. In contrast, external protections are justified as means to safeguard a group's practices and status from unfair disadvantages or cultural dominance imposed by the majority society.

Building on these distinctions, Kymlicka identifies three specific categories of minority rights: self-government rights (autonomy), multicultural rights, and special representation rights (Kymlicka, 1998, p. 9). These rights are all grounded in the liberal principle of individual autonomy and aim to ensure fair conditions for self-determination, cultural survival, and democratic participation.

However, Kymlicka's classification does not necessarily capture the full complexity of real-world situations. For instance, in countries like Japan, which historically have not embraced large-scale immigration or recognized themselves as multination states, the boundary between multination and polyethnic frameworks remains ambiguous. As a result, challenges persist in institutional design and policy development regarding how to recognize and accommodate diverse cultural identities in a context that lacks explicit multicultural policy foundations.

#### 4.1.3 Bhikhu Parekh: “politics of tolerance” emphasizing mutual transformation between cultures

In his seminal work, *Rethinking Multiculturalism: Cultural Diversity and Political Theory* (2000), British philosopher Bhikhu Parekh fundamentally criticizes Western political theory for implicitly treating Western values and lifestyles as “universal”. He points out that the “cultural premises” underlying what a state considers to be justice or equality are often unconsciously biased toward a single culture. For example, values such as freedom, rationality, autonomy, and progress originated from specific cultural traditions and may not necessarily have the same meaning for other cultures. Therefore, states should not unilaterally impose ostensibly universal values but rather listen to the unique value frameworks of diverse cultures and make value judgements in a relative and dialogical manner (Parekh, 2000). Parekh positions cultural diversity not as a “special problem within liberalism,” but as an essential challenge that demands a reconfiguration of political philosophy as a whole. In this sense, his theory differs from “cultural relativism” and instead proposes a vision of “intercultural universalism” that encompasses mutual criticism and the possibility of reconfiguration.

Parekh's central argument is that in a multicultural society, each culture should relativize itself in relation to others and transform itself through dialogue. In other words, the key to social integration lies not in unilateral “assimilation” but in “mutual transformation” (Parekh, 2000). In this process, it is necessary to critically examine each other's values and customs, accept partial revisions, and build a common public sphere.

He also argues that the state should not pretend to be culturally neutral, but should aim to be a “morally self-aware state” that guarantees the recognition and participation of diverse cultural groups (Parekh, 2000). This is a proposal for institutional and ethical change based on reflection on the past, when the state used neutrality as an excuse for substantive exclusion. Rather than focusing on specific groups such as indigenous peoples and immigrants, his argument is characterized by its focus on the general structure of cultural diversity and the principles of governance, and its theorization of the reconstruction of the relationship between the state, citizens, and cultures.

### 4.2 Multiculturalism policies

#### 4.2.1 Respect and recognition of cultural groups

The core of multiculturalism policy lies in respecting and recognizing the identities of groups with distinct cultural attributes such as religion, language, and lifestyle. This goes beyond mere tolerance and includes support through public institutions and budgets. For example, in the United Kingdom, policies such as mother tongue education, approval of religious ceremonies, and provision of places of worship have been implemented since the 1980s (Modood, 2007).

#### 4.2.2 The failure and criticism of multiculturalism

While multiculturalism has been justified in Canada, cautionary arguments have been raised regarding the separatist tendencies of minorities in Quebec and other regions (Bibby, 1990). In the early 2000s, voices pointing out the limitations of multiculturalism spread across European countries. This was due to a tendency for cultural groups to form closed communities and avoid engagement with the rest of society. Such trends were viewed as problematic, as they could lead to radicalization among some young people and serve as a breeding ground for homegrown terrorism. In various European countries, concerns about national integration and social cohesion have led to political reassessments of multicultural policies. In Germany, then Chancellor Angela Merkel remarked in 2010 that attempts to build a multicultural society had not achieved their goals, sparking renewed debates on integration models (The Guardian, 2010). In the United Kingdom, then-Prime Minister David Cameron similarly critiqued “state multiculturalism” in a 2011 speech, arguing for a stronger national identity and shared values (Government UK, 2011). These political framings, though controversial, reflect broader societal anxieties rather than empirically established causal relationships. More recently, Sweden's coalition government, supported by right-leaning parties including the Sweden Democrats, has adopted more restrictive immigration measures, citing challenges of integration and public sentiment (Ringstrom and Johnson, 2022).

#### 4.2.3 Direction for correction: integration policies and mutual education

In response to such criticism, many countries are shifting their policies toward striking a balance between “cultural recognition” and “social integration.” In the United Kingdom, “community cohesion” policies are being promoted, while Germany and Denmark are advancing “integration policies,” focusing on language education, employment support, and fostering mutual understanding (Vertovec and Wessendorf, 2010). The key to such policies lies not only in supporting minorities but also in holding the majority population responsible for understanding other cultures and engaging in dialogue. School education, community events, and media awareness campaigns are central to this approach.^13^

### 4.3 Comparison of Multiculturalism and Japanese “Tabunka-ky$\bar{\text{o}}$sei”

#### 4.3.1 Differences in ideology and structure

“Multiculturalism” in countries such as Canada, Australia, and the United Kingdom is based on the institutional recognition of cultural groups, with explicit provisions for the maintenance and respect of specific cultures in education, welfare, and language policies (Kymlicka, 2001; Parekh, 2000). In contrast, Japan's “tabunka-ky$\bar{\text{o}}$sei is an administrative term led by the Ministry of Internal Affairs and Communications that lacks a clearly defined philosophical concept and does not explicitly state that “cultural diversity contributes to the public interest,” thereby differing from multiculturalism (CLAIR, 2020). Japanese “tabunka-ky$\bar{\text{o}}$sei is not a concept that has been carefully thought out philosophically but a slogan reminiscent of the phrase “harmony is to be valued” that is said to have been established by Prince Sh$\bar{\text{o}}$toku in the early 7th century as the first article of Japan's first written constitution, “The Seventeen Article Constitution”. It cannot be said to define the rights of cultural groups or the meaning of coexistence. Given this nature, “tabunka-ky$\bar{\text{o}}$sei can be interpreted as a utilitarian term aimed at avoiding trouble and maintaining peaceful daily life. Furthermore, the ultimate goal of tabunka-ky$\bar{\text{o}}$sei seems to be adaptation to Japanese society, and it does not appear that the preservation of cultural diversity among cultural groups is regarded as an important right. As a result, “tabunka-ky$\bar{\text{o}}$sei seems to have been widely accepted as an abstract slogan on the same level as “everyone should get along” in general moral education through campaigns implemented by the government and educational institutions. A Japanese Sociologist Naoto Higuchi acknowledges a significant gap between tabunka-ky$\bar{\text{o}}$sei as a general idea and as a policy term. However, he states that “the concept of tabunka-ky$\bar{\text{o}}$sei itself is not meaningless; depending on how it is restructured, it can be made effective and serve as an ideological goal.” [Translated by author] (Takaya et al., 2019, p. 130).

#### 4.3.2 Policy trends for different types of minorities in Japan

Regarding indigenous peoples (Ainu in northern Hokkaido and Ryukyu in southern Okinawa), cultural promotion measures are being implemented, but there has been no apology or compensation for their historical oppression, nor has affirmative action been taken. In particular, regarding the Ainu people, the *Ainu Policy Promotion Act* (Act No. 16 of 2019) enacted in 2019 recognized the Ainu people for the first time as an “indigenous people,” and the law aims to promote policies to realize a society where the pride of the Ainu people is respected. However, under the *Hokkaido Former Indigenous People Protection Law*, which was in effect prior to the *Ainu Cultural Promotion Law* enacted in 1997, the derogatory term native (dojin, used since the Meiji era in the 19th century) was still in use (Siddle, 2014). To this day, the Japanese government has not issued an official apology for the discriminatory treatment of the Ainu people since the Meiji government's rule, and in this regard, its response can be considered insufficient in terms of multiculturalism.

Old-comers—mainly Zainichi Koreans, referring to Koreans and their descendants who either migrated from the Korean Peninsula during Japan's colonial rule (1910–1945) or were born in Japan to such families—are generally indistinguishable from Japanese people in appearance. Especially from the second generation onward, they are often regarded as effectively integrated into Japanese society, due in part to their native-level Japanese language proficiency. However, issues related to permanent residency status and the acquisition of Japanese nationality remain central to their situation.

New-comers—Brazilian, Filipino, Vietnamese and others—have been accepted since the 1990s as a necessary labor force for economic growth, but they have been treated as “tolerated” as “those who are expected to return to their home countries in the future”. “The Japanese government does not officially recognize these people as “immigrants”.^14^

#### 4.3.3 Education and policy implementation

In Japan, mother tongue education to nurture minority cultural identity relies on private international schools and ethnic schools (Korean schools, Brazilian schools, etc.). Public schools in Japan have taken measures such as assigning teachers to support minority students' learning, but public support for mother tongue education is limited to cultural introduction and international exchange programs. This is because the original purpose of tabunka-ky$\bar{\text{o}}$sei as a public policy in Japan is not to maintain the cultural identities of minorities from other countries, but rather to promote understanding of and exchange with Japanese people (Takaya et al., 2019, p. 106–128).

Overall, the theories of multiculturalism proposed by Taylor, Kymlicka, and Parekh provide a political philosophical basis for “recognition at the level of cultural units” (Taylor, 1994; Kymlicka, 2001; Parekh, 2000). However, Japan's tabunka-ky$\bar{\text{o}}$sei policies lack clarity regarding their ideal and philosophical foundations, and they appear to be weak in their connection to political philosophy and rights discourse, instead leaning heavily toward pragmatism.

## 5 Evaluation of tabunka-ky$\bar{\text{o}}$sei based on Honneth's recognition theory

The characteristics and challenges of “tabunka-ky$\bar{\text{o}}$sei in contrast to “multiculturalism” have now been examined. This raises the question of whether the concept and policy of tabunka-ky$\bar{\text{o}}$sei are merely immature and inferior to multiculturalism and whether this has any positive significance. To address this issue, the following section examines Axel Honneth's theory of recognition and evaluate Japanese tabunka-ky$\bar{\text{o}}$sei from the perspective of its key elements.

### 5.1 Honneth's theory of recognition

Axel Honneth shares common ground with Charles Taylor, who defends multiculturalism, in his discussion of Hegel's theory of recognition (Taylor, 1994; Honneth, 1996). However, Honneth refers to the three-stage theory of recognition developed by the young Hegel during his Jena period, and based on this, distinguishes the nature of recognition as follows: (1) recognition of “love” in the intimate sphere, (2) “legal” recognition as equal citizens, and (3) “social” recognition in society commensurate with one's contributions. These develop sequentially, with each preceding stage of recognition serving as the foundation for the next, and through recognition struggles mediated by communication, the scope of recognition expands (Honneth, 1996, 92-135).

Honneth's definition of “recognition” goes beyond mere evaluation or agreement, referring to the positive affirmation of one's existence by others.

By recognition I mean the social acknowledgment of an individual's worth, as a person capable of making claims in a community of equal moral standing. Recognition is the very process through which people become fully integrated into social life, where their capacities and uniqueness are recognized and validated by others (Honneth, 1996, p. 127).

Recognition is “a mutual subjective relationship in which the existence of one subject is affirmed by another, enabling the subject to relate to itself in a positive manner” (Honneth, 1996, p. 92). Furthermore, such recognition relations are more fundamental than conflicts over economic interests or resource distribution, and Honneth emphasizes that economic struggles themselves ultimately boil down to conflicts over recognition (Honneth, 2007).

The domains of recognition do not exist independently but are interrelated, and the acquisition or lack of recognition in one domain has a ripple effect on other domains. For example, recognition of love in the intimate sphere forms the basis of social self-consciousness and determines the possibility of receiving legal and social recognition (Honneth, 1996, p. 95–99).

Honneth builds on Hegel's “Jena period” theory of recognition in his youth, while drawing on Habermas's framework of dialogical rationality, to develop a “post-metaphysical” conception in which the formation of social recognition progresses not through violent struggle but through consensus-building in public communication (Honneth, 1996; Habermas, 1996). Furthermore, Honneth refers to George Herbert Mead's social psychology to emphasize the importance of social recognition as a fundamental condition for the formation of personal identity (Honneth, 2007). In Mead's theory, the self is formed within the “generalized other,” which is the internalized gaze of others, and social recognition serves as the foundation for this self-formation (Mead, 1934).

Furthermore, based on Mead's framework, Honneth argues that each sphere of recognition not only moves unidirectionally toward higher spheres, but that recognition in higher spheres (e.g., social recognition) also influences lower spheres (e.g., the intimate sphere) through feedback (Honneth, 1996, p. 130). Therefore, he argues that the lack of social recognition has a negative impact on the recognition of love in the intimate sphere, that is, family relationships and self-esteem.

### 5.2 Significance of Honneth's theory of recognition

#### 5.2.1 The concept of recognition

Recognition refers to the acceptance of one's existence and identity by others, which has a profound impact on one's self-evaluation and self-esteem beyond mere material benefits. A lack of recognition may lead to psychological distress, social exclusion, and even alienation from social relationships (Honneth, 1996). For instance, when people voluntarily pick up trash in their neighborhoods, their actions can be interpreted not only as altruism but also as an effort to be recognized as contributing members of the community. From this perspective, unilateral support for cultural minorities—when it frames them merely as vulnerable recipients of aid—can unintentionally undermine their dignity and hinder their desire to be acknowledged as equal members of society. Social recognition requires not only compassion but also acknowledgment of minorities' capacity to contribute. When individuals from minority backgrounds engage in community service or other forms of social contribution, it strengthens their recognition within society as citizens with equal status and capabilities, rather than as passive beneficiaries.

#### 5.2.2 Distinction between three areas of recognition

Honneth's distinction between three domains of recognition—love, legal recognition, and recognition in the social sphere—is significant in that it enables analysis of social recognition in addition to administrative and legal recognition (Honneth, 1996). This is similar to the achievement of Hannah Arendt, who, in The Human Condition, introduced the category of “action” in addition to “labor” and “work” as forms of human existence (Arendt, 1958). In other words, Honneth's social recognition serves as a perspective for evaluating the significance of citizens' social participation. For example, in a multicultural society, there may be cases where members of minority groups have certain legal recognition (citizenship) but lack social recognition in the social sphere, or conversely, cases where non-regular residents who lack legal recognition obtain social recognition through community participation.

Moreover, Honneth's theory of recognition offers a complementary framework for reinterpreting T.H. Marshall's classic theory of citizenship (Marshall, 2021). Marshall describes a historical development of citizenship progressing from civil rights (18th century), to political rights (19th century), and finally to social rights (20th century). While this trajectory is often viewed as the expansion of legal entitlements, it can also be interpreted as a deepening of societal recognition. Marshall's stages and Honneth's categories align as follows: civil and political rights correspond to the sphere of legal recognition, fostering self-respect, whereas social rights resonate with the dimension of social solidarity, enhancing self-esteem. Importantly, legal recognition continues to play a role even in the realization of social rights, underscoring the layered and overlapping nature of recognition across institutional domains. Taylor and Kymlicka's multiculturalism theory emphasizes the “double guarantee” of legal recognition for cultural groups and civic recognition for the state, and justifies this (Taylor, 1994; Kymlicka, 1995). However, Honneth's theory stresses that social recognition from the whole society through public social participation is indispensable, beyond the legal recognition of collective identity. This is compatible with Parekh's argument in that it recognizes the necessity of engagement with society as a whole and the risk of community separation (Parekh, 2000).

This three-stage theory of recognition is useful not only for analyzing individual cases but also for comparing recognition structures in different multicultural societies, i.e., for understanding the differences in institutional designs and ideologies in countries such as Canada, Germany, and Japan. Furthermore, Honneth's perspective that the three domains are interrelated contributes to a dynamic analysis of whether the enhancement of one domain (e.g., legal recognition) leads to the enhancement of other domains (e.g., social evaluation). In this way, the theory of recognition serves as a “framework for recognition and organization” that provides a theoretical foundation for everything from describing the current situation to identifying issues and designing institutions.

While Honneth emphasizes that social recognition is grounded in individuals' perceived contributions to society, it is crucial to distinguish his notion of “achievement” from a narrow meritocratic or economic perspective. Rather than referring solely to professional or productive success, Honneth's concept of achievement includes diverse forms of social contribution, such as caregiving, volunteerism, or cultural engagement, provided they are valued within a given social context (Honneth, 1996, p. 121–125).^15^

#### 5.2.3 Presentation of norms: in light of the issue of isolationism

Honneth's argument regarding the necessity of social recognition is also mentioned in Taylor's (1994) examination of The Politics of Recognition, but it raises the question of whether recognition from a more inclusive society is unnecessary in cases where isolated communities such as the Amish in the United States are satisfied with recognition within their own group.

Furthermore, it is theoretically possible to treat such communities as objects of “tolerance” from an inclusive society. However, because this carries the risk of severing public engagement, social recognition is indispensable for (Honneth 1996). In this way, treating social recognition as a “normative theory” makes it possible to visualize imbalances and deficiencies in recognition, thereby providing a normative theoretical tool that can give direction to future social policies.

### 5.3 Critical perspectives on Honneth's theory

#### 5.3.1 Rejection of a unilinear historical view of recognition struggles as progress

Honneth, following Hegel, tends to view the struggle for recognition in society as a process of historical progress that is, the expansion of freedom (Honneth, 1996). Social friction and conflict are said to expand the realm of recognition and lead to the realization of more universal freedom and autonomy. However, this framework may not necessarily apply to current or future societies. In modern society, the concepts of freedom and autonomy are becoming ambiguous through new forms of domination and governance, such as nudge theory (Thaler and Sunstein, 2008) and technology (e.g., algorithmic preference guidance). Even when individual *free choice* appears to be respected on the surface, it is often the result of unconscious guidance and cannot be considered true autonomy in the original sense. Therefore, a skeptical perspective is necessary regarding Honneth's linear progression from - struggle for recognition → institutional reform → advancement of freedom. As a result, the value of Honneth's theory is limited to demonstrating the *probability of mutual recognition*, and it does not necessarily mean that the struggle for recognition will be realized and institutional progress will follow.

#### 5.3.2 Flexible treatment and comparability of the three categories of recognition

Honneth's three-category classification (love, legal recognition, solidarity) allows for a careful understanding of the structure of recognition, but in actual application, one should not be overly fixated on this classification (Honneth, 1996). This is because, in social phenomena, multiple domains of recognition often overlap and influence one another, making clear distinctions difficult in many cases. Furthermore, in a comparative social context, there are diverse forms of recognition depending on the country and system, such as cases where *legal status is guaranteed* but *social esteem* is lacking, or cases where “human rights can be asserted but recognition is not granted at the national level.” For this reason, rather than strictly applying the classification, it is desirable to apply the conceptual framework flexibly.

### 5.4 Japan's Tabunka-ky$\bar{\text{o}}$sei from the perspective of Honneth's recognition theory

#### 5.4.1 Evaluation of the concept of Tabunka-ky$\bar{\text{o}}$sei

In this section, selected local practices are illustratively introduced to explore how recognition theory can illuminate the challenges and potential of Japan's tabunka-ky$\bar{\text{o}}$sei.^16^ The concept of tabunka-ky$\bar{\text{o}}$sei promoted by the Japanese government primarily focuses on differences in nationality, ethnicity, language, and culture. Policies formulated by local governments and the Ministry of Internal Affairs and Communications have been implemented with a focus on coexistence with foreign residents and immigrants.^17^ However, specific goals vary depending on the minority group in question. For example, the Ainu are targeted for cultural exchange and awareness programs, with an emphasis on promoting traditional culture. Regarding “legal recognition,” it is granted within the framework of residence status. For groups such as Zainichi Koreans (Korean ethnic communities residing in Japan) and technical trainees, the focus is on cross-cultural communication and Japanese language education, and policies aimed at preserving and ensuring cultural identity are not a priority. Within the framework of Honneth's recognition theory, the fundamental goal of tabunka-ky$\bar{\text{o}}$sei can be explained through the “social recognition” of minority groups in Japan.

Movement in this direction is evident in the revision of the 2006 *Tabunka-ky*$\bar{\text{o}}$*sei Promotion Plan* implemented in September 2020 (Ministry of Internal Affairs and Communications, 2020). The revised plan, updated in line with the diversification of foreign residents and changes in Japan's socio-economic situation, outlines the following four strategic priorities: (1) Building a “new normal” through the promotion of a diverse and inclusive society; (2) Promoting the contribution of foreign residents to regional revitalization and globalization; (3) Promoting the active participation of foreign residents in local communities and diversifying the number of people involved; and (4) Establishing a decentralized framework for accepting foreign residents to prevent excessive concentration in urban areas. This revised plan supports the employment and social integration of international students while encouraging local governments to engage with foreign residents not merely as “objects of support” but as “active partners” in community development. These revisions signify a policy shift from one-way support to mutual engagement and recognition, aiming to expand the concept of “tabunka-ky$\bar{\text{o}}$sei from a framework of cultural tolerance to one of inclusive participation and shared responsibility in community governance (Ministry of Internal Affairs and Communications, 2020).

#### 5.4.2 Challenges in tabunka-ky$\bar{\text{o}}$sei policies

Japan's tabunka-ky$\bar{\text{o}}$sei policies are implemented through collaboration among local governments, the Ministry of Internal Affairs and Communications, the Ministry of Health, Labor and Welfare, NPOs, and other relevant actors, based on the number and characteristics of foreign residents in each region. The revised plan outlines directions for addressing these challenges, and while some progress has been made in the implementation of tabunka-ky$\bar{\text{o}}$sei policies to date, structural challenges remain.

First, while physical coexistence exists, there is a lack of psychological and social interaction with “invisible others.” In particular, prejudice, discriminatory attitudes, and indifference hinder the building of mutual understanding and trust. In response to this situation, staff members of international exchange associations and NPOs in various regions are actively working with limited resources, but cuts in financial support from the national government and a shortage of human resources are placing serious constraints on the continuation of activities and the treatment of staff (e.g., SPIRA in Saga Prefecture and HICE in Hamamatsu City; see institutional overview in Section 2). This is not unrelated to the fantasy of a “single-ethnic nation” that is deeply rooted in Japanese society. Japan's immigration policy has been consistently selective and restrictive, and despite professing to promote tabunka-ky$\bar{\text{o}}$sei, there are virtually no substantive “institutional integration policies.” As a result, policy resources and implementation systems to support tabunka-ky$\bar{\text{o}}$sei are inadequate, creating the risk of a vicious cycle of policy indifference → marginalization of minorities → self-fulfilling prophecies.

Regarding public recognition and institutional responses to the protection of ethnic identity among minority groups, “integration” into the host society (i.e., Japanese society) is often taken for granted, while the preservation of individual cultural traditions and languages is often viewed as a secondary issue. In fact, while there are cases in which teachers proficient in foreign languages provide individual support in public schools, this is not systematically implemented as a policy, unlike Canada's multilingual education policy. As an exception, in $\bar{\text{O}}$izumi Town, Gunma Prefecture, several Brazilian schools offer Portuguese language education to support children and residents who may return to Brazil (Jōmō Shimbunsha, 2022). For other language groups, however, such as American, French, and Korean schools are private institutions, and receive only limited support from public authorities.

With regard to education, the “right to education” as stipulated in the Universal Declaration of Human Rights and the Convention on the Rights of the Child is not fully guaranteed for foreign children in Japan, and the Japanese compulsory education system does not apply to foreigners. Furthermore, since children's residence status is linked to that of their parents, their enrollment in school tends to be unstable. The failure to fulfill the “right to education” for foreign children not only poses functional issues that hinder their social integration but also raises universal human rights concerns, specifically regarding “legal recognition.” Such institutional vulnerabilities pose serious risks of adversely affecting children during their identity formation period. In particular, parents' feelings of social alienation and stress can spread to the intimate sphere of the family, potentially adversely affecting children's self-esteem and social participation through parent-child relationships.

According to Honneth's recognition theory, this can be interpreted as a structural vicious cycle in which the lack of solidarity, as recognition in the social sphere “recognition in the intimate sphere.” Furthermore, when foreign children are unable to maintain their native language and cultural heritage, they face the risk of communication breakdown with their parents and reversal of social status, which may make close communication between parents and children difficult. As a result, the home, which should function as a safe place, may fail to provide a safe space, potentially leading to problems with “approval in the intimate sphere” in parent-child relationships. Furthermore, these family circumstances may compel children to hide their foreign roots or try to make them less noticeable, which could pose a risk to “social recognition.” (Takaya et al., 2019, p. 118).

Regarding language ability, according to the Ministry of Education, Culture, Sports, Science and Technology (2024) *Survey on the Acceptance of Children and Students Requiring Japanese Language Education*, the number of children and students in public elementary, junior high, and high schools who require Japanese language instruction reached 69,123 as of May 2023. This represents an increase of 10,816 students (18.6%) compared to the previous 2021 survey, and nearly double the number from 2008. Of these, 57,718 were foreign nationals (a 21.2% increase from 2021), while 11,405 were Japanese nationals, including children of returnees and those with foreign-born parents (a 6.7% increase). Among students identified as needing language support, 90.4% of foreign nationals and 86.6% of Japanese nationals received instruction with special consideration from schools. However, these support rates slightly declined compared to the previous survey, suggesting that schools are struggling to keep up with the rising number of students in need. Moreover, while 90.3% of Japanese language learners proceed to upper secondary school (a slight increase from 89.9% in 2021), this remains significantly lower than the overall transition rate of 99.0%. The dropout rate among such students is 8.5%, a sharp contrast to the national average of 1.1%. University enrollment among these students stands at 46.6%, compared to 75.0% of all high school graduates, while the non-regular employment rate among those who do find work is 38.6% (vs. 3.1% nationally). These disparities highlight serious structural barriers to educational attainment and social mobility for students requiring Japanese language instruction. Furthermore, despite the Japanese government's emphasis on the importance of Japanese language education in daily life in language support programs for adult immigrants, the level of Japanese required for their daily lives appears to be far from sufficient for employment purposes. Language education for foreign workers, such as technical trainees, who are essentially immigrants, is inadequate because it is based on the assumption that they will return to their home countries after a few years. It has been pointed out that Japan's language support is far weaker than language training programs aimed at integrating immigrants into the labor market in countries such as Germany, France, Australia, the Netherlands, and Denmark (OECD member countries) (Takaya et al., 2019, p. 33–34; OECD, 2018; MIPEX, 2020). These circumstances are considered to pose serious risks to “social recognition” achieved through language-mediated communication and mutual understanding, as well as the related “recognition within the intimate sphere”.

#### 5.4.3 Advanced initiatives addressing challenges in tabunka-ky$\bar{\text{o}}$sei

As mentioned above, current tabunka-ky$\bar{\text{o}}$sei policies face numerous challenges. Additionally, since these policies vary in content depending on the number of foreign residents and regional characteristics in each municipality, it is difficult to evaluate the status of efforts to address these challenges in a generalized manner. However, in the area of “social recognition,” which is the primary focus of tabunka-ky$\bar{\text{o}}$sei policies, some advanced initiatives are also emerging.

As a good example of promoting exchange between foreign residents and local residents and supporting Japanese language education, we would first like to highlight Hamamatsu City in Shizuoka Prefecture, which is working to promote the participation of foreign residents in the local community by shifting the perception of foreigners from “people who need support” to “equal residents.” In Hamamatsu City, which has a high foreign resident population and proportion, the Hamamatsu International Center for Exchange (HICE), a public interest incorporated foundation, has established multilingual websites and consultation desks for foreigners, and implemented awareness campaigns including resident training sessions and interview booklets (based on an interview with HICE representatives on April 22, 2025). Hamamatsu City is the only municipality in Japan officially recognized as an “Intercultural City” by the Council of Europe (Council of Europe, 2008), and Oizumi Town's initiatives are clearly aligned with this direction. Oizumi Town in Gunma Prefecture, which has a large foreign population similar to Hamamatsu City and the highest percentage of foreign residents in Japan, has been accepting Brazilian Japanese as necessary labor for local industries since the early 1990s. The Oizumi Town Office Multicultural Collaboration Division and the Oizumi International Exchange Association have been at the forefront of implementing Japanese language classes for foreign residents and actively supporting their participation in community activities. Oizumi Town, with its large population of Japanese Brazilians, actively hosts events such as the “Samba Festival” and “World Gourmet Alley,” which feature professional performers from Brazil. These events serve as a platform for cultural exchange between minority groups and local residents (Jōmō Shimbunsha, 2022; based on interviews with $\bar{\text{O}}$izumi Town Hall staff and a member of the $\bar{\text{O}}$izumi International Association on April 23, 2025). The Council of Europe (2008) has been promoting *The Intercultural Cities Programme (ICC)* as a model for multicultural societies since 2008, aiming to actively utilize the cultural diversity brought by immigrants and minority groups to revitalize cities and foster creativity. Even in areas where the number and proportion of foreign residents are not high, there are regions that are actively promoting tabunka-ky$\bar{\text{o}}$sei. For example, in Saga Prefecture, the Saga Prefectural Multicultural Promotion Division and the Saga Prefecture International Exchange Association (SPIRA) are at the forefront of promoting community-based tabunka-ky$\bar{\text{o}}$sei, providing Japanese language education support and consultation services in three formats: community-based, corporate-based, and online. Additionally, the organizers create opportunities for residents to interact directly through “town meetings.” (based on an interview with staff members of the Saga Prefectural Multicultural Promotion Division on May 19, 2025).

Tabunka-ky$\bar{\text{o}}$sei policies are also being promoted by entities other than local governments. The NPO “Multicultural Center Tokyo” in Tokyo operates a paid free school four days a week, offering Japanese language education and supplementary lessons on the curriculum taught in Japanese middle and high schools (Multicultural Center Tokyo, 2025). The center prioritizes academic performance improvement, fostering self-esteem, and promoting social interaction (based on an interview with center staff on April 24, 2025).

The tabunka-ky$\bar{\text{o}}$sei initiatives of these local governments and NPOs are significant in that they have the potential to bring positive feedback to intimate areas by promoting the realization of “social recognition,” which is a key component of Honneth's recognition theory. For example, foreign children and students who are unfamiliar with Japanese are at a higher risk of bullying and isolation in school life, and in some cases, may face difficulties in their daily lives. As globalization progresses, English is useful as a common international language, but Japanese is the foundation of daily life in Japan, and its acquisition is indispensable. From this perspective, enhancing Japanese language education is essential, and multicultural education that addresses prejudice and discrimination is considered a meaningful policy for building the foundation of social recognition.

#### 5.4.4 The concept and potential of Tabunka-ky$\bar{\text{o}}$sei

This section has examined how selected local practices in Japan, when viewed through the lens of Honneth's theory of recognition, exemplify both the strengths and the limitations of tabunka-ky$\bar{\text{o}}$sei. In light of the innovative initiatives undertaken by local governments and NPOs, these policies hold certain potential and prospects despite the various challenges already mentioned. For example, continuing and strengthening existing measures such as Japanese language education, intercultural understanding education, and promoting resident exchanges could expand social recognition of cultural minorities and reduce barriers between cultural groups. This is also important from the perspective of preventing the “ghettoization” of specific ethnic groups. In the legal domain, it is necessary to guarantee universal human rights such as the right to education within the framework of residence status. However, policies that promote changes in civic awareness through multicultural education and intercultural exchange are also important in terms of fostering understanding and support for the protection of the rights of foreign residents. The asymmetry in legal status between nationals and non-nationals is an unavoidable issue within the framework of national sovereignty, but even if attempts are made to equalize legal status through naturalization or the introduction of multicultural citizenship, there is a risk that nominal equality will not lead to substantive integration if social recognition is lacking. In this regard, in a multicultural society based on the framework of national sovereignty, efforts to build social recognition within the institutional framework are essential for both the host society and minority groups. From this perspective, the concept and policies of tabunka-ky$\bar{\text{o}}$sei are considered to have significance and potential for realization. Furthermore, the realization of solidarity, understood as recognition in the social sphere, is considered to contribute to the enhancement of recognition in the intimate sphere and self-respect, which are inseparable from it. For example, when foreign residents contribute to local communities, this leads to social recognition, both of which in turn stabilizes recognition relationships within intimate circles such as family relationships and further promotes social participation, creating a virtuous cycle.

## 6 Conclusion

The first significant analysis in this paper is that Japan's tabunka-ky$\bar{\text{o}}$sei has characteristics that differ ideally and institutionally from multiculturalism, as demonstrated through comparison. The second finding is that by employing Axel Honneth's recognition theory, this study has systematically organized the characteristics and challenges of Japan's tabunka-ky$\bar{\text{o}}$sei policies and provided a framework for evaluating them. This makes it possible to restructure policies that had previously been vaguely understood and provide a basis for future policy planning and public debate.

In particular, Honneth's theory, which divides recognition into three stages-*intimate sphere, legal sphere*, and *social sphere*-provides a theoretical tool for comparing and analyzing cultural minority support measures and integration policies, which had previously been discussed separately, within a common framework (Honneth, 1996). This perspective highlights the importance of *solidarity* as a form of social recognition in a multicultural society and is similar to Smith's argument in *The Theory of Moral Sentiments* that a *fair observer* is formed within the subject through feedback not only from within the cultural group but also from society as a whole (Smith, 2002).

This perspective could also serve as an alternative to the pitfalls of multiculturalism in Europe, which, by overly emphasizing respect for ethnic communities, has led to ghettoization and social isolation (e.g., the home-grown terrorism noted by Merkel and Cameron) (Vertovec and Wessendorf, 2010). Based on Honneth's theory, social recognition is linked to intimacy and legal recognition, and it can be logically explained that promoting social engagement and dialogue across the entire society opens the way to a stable society of coexistence (Honneth, 2008).

The danger that a lack of social recognition could be self-fulfilling and destroy social bonds echoes the warnings of (Smith 2002), (Durkheim 2014), and (Tocqueville 2000). In modern multicultural nations, social stability cannot be guaranteed by legal and administrative frameworks alone; a network of mutual recognition and empathy across society as a whole is an indispensable element. The absence of recognition can become a *silent killer* that quietly erodes social order.

Tabunka-ky$\bar{\text{o}}$sei in Japan has thus far relied on grassroots initiatives such as *exchange* and *mutual understanding*. However, with the increasing number of foreign residents and the progression of cultural diversity, it is now necessary to address more fundamental questions such as “What is ky$\bar{\text{o}}$sei(coexistence, co-living)?”, “Whose values form the public sphere?”, and “To what extent should differences be tolerated?” The tension surrounding Kurdish illegal refugees in Warabi City, Saitama Prefecture, and the demand for burial following religious and cultural practices among Islamic foreign residents are examples of situations that require responses to cultural differences that were not previously anticipated, and they are significant as a starting point for future discussions (Yomiuri Shimbun, 2023).

Going forward, it will be necessary for society as a whole to engage in deep deliberation not only on temporary measures and exchange programs, but also on the ideology, institutions, and education appropriate for a multicultural society. Honneth's theory of recognition can be positioned as an effective framework for promoting future-oriented institutional design and social dialogue (Honneth, 1996).

## Data Availability

The raw data supporting the conclusions of this article will be made available by the authors, without undue reservation.

## Footnotes

Edited by: Gottfried Schweiger, University of Salzburg, Austria

Reviewed by: Xiao Wang, Suzhou Lunhua Education Group, China Emil A. Sobottka, Pontifical Catholic University of Rio Grande do Sul, Brazil

## Footnotes

1 A Japanese sociologist Keizo Yamawaki acknowledges the difficulty of translating the Japanese term tabunka ky$\bar{\text{o}}$sei into English. Nevertheless, he declares his own usage of the term: referring to a tabunka ky$\bar{\text{o}}$sei society as an “intercultural society,” the creation of such communities as “intercultural community building,” and tabunka ky$\bar{\text{o}}$sei itself—alongside the original Japanese term—as “intercultural cohesion” (CLAIR, 2020).

2 Hamamatsu City is located in Shizuoka Prefecture, central Japan, Hamamatsu is known for its significant foreign population, particularly of Brazilian descent. The city began promoting tabunka-ky$\bar{\text{o}}$sei policies in the 1990s due to the influx of foreign workers in the manufacturing sector.

3 $\bar{\text{O}}$izumi town is situated in Gunma Prefecture, about 80 km northwest of Tokyo, $\bar{\text{O}}$izumi has one of the highest proportions of foreign residents in Japan, particularly Japanese-Brazilian and Peruvian communities. The town is recognized for its long-standing commitment to multicultural initiatives since the early 1990s.

4 Multicultural Center Tokyo is a certified NPO based in Tokyo that supports children with foreign roots through language and academic education. Originating after the 1995 Hanshin-Awaji Earthquake, it became independent in 2006. The center promotes tabunka-ky$\bar{\text{o}}$sei via its “Tabunka Free School” and advocacy for inclusive education and human rights.

5 Saga Prefecture is located in Kyushu, southwestern Japan. Although it has a smaller foreign population compared to Hamamatsu and $\bar{\text{O}}$izumi, it has developed regionally rooted multicultural policies.

6 Japan's Technical Intern Training Program (TITP) is a state-managed scheme introduced in 1993, officially aimed at transferring skills to workers from developing countries. In practice, however, it has often been criticized as a form of low-wage labor importation under the guise of training, with limited legal protection and restricted freedom of job change. Participants are expected to return to their home countries after completing a fixed-term contract, usually 3 to 5 years.

7 Since there is no official English version of this document from the Ministry of Internal Affairs and Communications, it is unclear how the Ministry intends to translate tabunka ky$\bar{\text{o}}$sei into English.

8 This policy was revised in 2020 and the revised version emphasizes building a “new normal” society that is inclusive and diverse, where foreign residents are recognized as full members of the community (Ministry of Internal Affairs and Communications, 2020). It promotes multilingual information services, community-based Japanese language education, and ICT use for communication and crisis response. Additionally, it encourages active participation of foreign residents in revitalizing local communities and supports their integration through employment, entrepreneurship, and local engagement.

9 The concept of “tabunka-ky$\bar{\text{o}}$sei education” proposed by the Fundamental Education Policy for Foreign Residents in Yokohama City (1991) and the Osaka Prefecture Foreigners' Education Research Council, which was established in 1992, aims to foster an understanding and respect for ethnic cultures among the Korean residents in Japan. This approach was oriented toward promoting an attitude of respect for cultural diversity, distinct from the direction of assimilation (Takaya et al., 2019, p. 111).

10 For instance, (Lie 2001) and (Befu 2001) suggest that Japan's emphasis on homogeneity and group harmony can be seen as being shaped by cultural traditions that include Confucian elements. However, such arguments remain interpretive and should be approached with nuance and contextual awareness.

11 In Japan, nationality acquisition is based not on “jus soli” (birthright) but on “jus sanguinis” (bloodline). While naturalization is possible, applicants are typically required to have more than five years of continuous residence, good conduct, financial stability, and Japanese language proficiency. The application process is often bureaucratically burdensome and informally assimilationist, resulting in a comparatively high threshold for acquiring citizenship (e.g., OECD, 2021, 2024; International Organization for Migration (IOM), 2022).

12 Warabi City in Saitama Prefecture has one of the highest concentrations of Kurdish residents in Japan, many of whom arrived as asylum seekers. In recent years, tensions have risen between Kurdish communities and local residents, drawing national attention to issues of refugee policy and multicultural integration.

13 The author agrees with this direction and, applying Adam Smith's theory of sympathy found in *The Theory of Moral Sentiments* (1759), emphasizes the necessity of a “sense of kinship” throughout society through ‘horizontal relationships' in which people with cultural differences empathize with one another, in addition to vertical relationships based on hierarchical institutional guarantees (Ishimatsu, 2017). Smith's “sympathy,” or “the ability to feel what another feels,” can provide the basis for coexistence not only in institutional design but also in ethical attitudes. “Man naturally desires, not only to be loved, but to be lovely.” - Adam Smith, *The Theory of Moral Sentiments*, Part III, Chapter II. The establishment of horizontal relationships based on such empathy is the key to promoting relational equality and social trust across the lines of majority and minority.

14 As a characteristic of Japan's immigration policy, the policy of not accepting “unskilled laborers” has been maintained for many years. However, foreign workers have been accepted through the so-called “side door” under the guise of engaging in technical internships or entertainment work. The reason for such seemingly contradictory policies has been pointed out as an attempt to reconcile the anti-immigration far-right forces with the economic sectors seeking labor (Takaya et al., 2019, p. 23).

15 However, this approach raises normative concerns regarding those who, due to structural inequalities or personal circumstances, may lack opportunities to visibly “achieve.” As (Fraser 2000) argues, an inclusive theory of recognition must account not only for performative recognition but also for the unconditional respect for individuals' dignity. Thus, while Honneth's framework allows for a nuanced understanding of recognition through contribution, it must be supplemented with a broader ethics of inclusion to avoid the marginalization of the less visible or “unproductive”.

## References

[B1] Arendt H. (1958). The Human Condition. Chicago, IL: University of Chicago Press.

[B2] Arudou D. (2015). Embedded Racism: Japan's Visible Minorities and Racial Discrimination. Lanham, MD: Lexington Books.

[B3] Asahi Shimbun (2022). Only Seven Cemeteries in Japan Accept Muslim Burials. The Asahi ShimbunAsia & Japan Watch. Available online at: https://www.asahi.com/ajw/articles/14608984 (Accessed August 13, 2025).

[B4] Befu H. (2001). Hegemony of Homogeneity: An Anthropological Analysis of Nihonjinron. Tokyo: Trans Pacific Press.

[B5] Bibby R. W. (1990). Mosaic Madness. Boston, MA: Stoddart Pub.

[B6] Cabinet Secretariat (2019). Ainu seisaku suishinh$\bar{\text{o}}$ *[Act on the Promotion of Ainu Policy]*. Available online at: https://www.kantei.go.jp/jp/singi/ainusuishin/ (Accessed August 13, 2025).

[B7] CLAIR (2020). Tabunka-ky$\bar{\text{o}}$*sei 2.0 no jidai: Dai 30-kai “Tabunka-ky*$\bar{\text{o}}$*sei” no eigoyaku wa d*$\bar{\text{o}}$ *shitara yoi ka [The Era of Multicultural Coexistence 2.0: Vol. 30 “How Should We Translate ‘Tabunka-ky*$\bar{\text{o}}$*sei' into English?”]*. Available online at: https://www.clair.or.jp/tabunka/portal/column/contents/114785.php (Accessed August 13, 2025).

[B8] Council of Europe (2008). Intercultural Cities: Governance and Policies for Diverse Communities. Available online at: https://www.coe.int/en/web/interculturalcities/ (Accessed August 13, 2025).

[B9] Durkheim E. (2014). The Division of Labor in Society. New York, NY: Free Press. (Original work published 1893).

[B10] Fraser N. (2000). Rethinking recognition. New Left Rev. 107–120.

[B11] Government UK (2011). PM's Speech at Munich Security Conference. Available online at: https://www.gov.uk/government/speeches/pms-speech-at-munich-security-conference (Accessed August 13, 2025).

[B12] Habermas J. (1996). Between Facts and Norms: Contributions to a Discourse Theory of Law and Democracy. Cambridge, MA: MIT Press.

[B13] Hamamatsu City (2023). Tabunka ky$\bar{\text{o}}$*sei shisaku [Multicultural Coexistence Policy]*. Available online at: https://www.city.hamamatsu.shizuoka.jp/ (Accessed August 13, 2025).

[B14] Hamamatsu Foundation for International Communication and Exchange (HICE) (2025). Official Website. Available online at: https://www.hi-hice.jp/ja/ (Accessed August 13, 2025).

[B15] Honneth A. (1996). The Struggle for Recognition: The Moral Grammar of Social Conflicts. Cambridge: Polity Press.

[B16] Honneth A. (2007). Disrespect: The Normative Foundations of Critical Theory. Cambridge: Polity Press.

[B17] Honneth A. (2008). Reification: A New Look at an Old Idea. Oxford: Oxford University Press.

[B18] Immigration Services Agency of Japan (2024). Tokutei gin$\bar{\text{o}}$ *zairy*$\bar{\text{u}}$*shani kansuru t*$\bar{\text{o}}$*kei [Statistics on Specified Skilled Workers]*. Available online at: https://www.moj.go.jp/isa/content/930004452.pdf (Accessed August 13, 2025).

[B19] International Organization for Migration (IOM) (2022). World Migration Report 2022. International Organization for Migration. Available online at: https://publications.iom.int/books/world-migration-report-2022 (Accessed August 29, 2025).

[B20] Ishihara K. (2004). Tabunka ky$\bar{\text{o}}$sei to Nihon shakai [Multicultural coexistence and Japanese society]. Int. Sociol. 1-20. Available online at: https://www.isfj.net/articles/2004/kokusai_ishihara.pdf (Accessed August 13, 2025).

[B21] Ishimatsu H. (2017). Sympathy as a Foundation of Multicultural Society: A Dialogue with Adam Smith. Rome: Edizioni di Storia e Letteratura.

[B22] Iwate Prefectural Government (2016). Iwate Prefecture Multicultural Coexistence Plan. Available online at: https://www.pref.iwate.jp/ (Accessed August 13, 2025).

[B23] Jōmō Shimbunsha (2022). Sanba no machi sorekara: Gaikokujin to tomo ni ikiru Gunma $\bar{\text{O}}$*izumi [The Samba Town and Beyond: Living Together with Foreigners in* $\bar{\text{O}}$*izumi, Gunma]*. J$\bar{\text{o}}$m$\bar{\text{o}}$ Shimbunsha.

[B24] Kojima S. (2023). To bury or not to bury: Muslim migrants and the politics of funerary rights in contemporary Japan. Asia Pacif. J. Jpn. Focus. Available online at: https://apjjf.org/2023/11/kojima (Accessed August 13, 2025).

[B25] Kymlicka W. (1995). Multicultural Citizenship: A Liberal Theory of Minority Rights. Oxford: Clarendon Press.

[B26] Kymlicka W. (1998). Finding Our Way: Rethinking Ethnocultural Relations in Canada. Oxford: Oxford University Press.

[B27] Kymlicka W. (2001). Politics in the Vernacular: Nationalism, Multiculturalism, and Citizenship. Oxford: Oxford University Press.

[B28] Kymlicka W. (2002). Contemporary Political Philosophy: An Introduction, 2nd Edn. Oxford: Oxford University Press.

[B29] Lie J. (2001). Multiethnic Japan. Cambridge, MA: Harvard University Press.

[B30] Mainichi Shimbun (2023). Warabi-shi de Kurudo-kei j$\bar{\text{u}}$*min to chiiki j*$\bar{\text{u}}$*min no tairitsu [Conflict Between Kurdish and Local Residents in Warabi City]*. Available online at: https://mainichi.jp/articles/20230610 (Accessed August 13, 2025).

[B31] Marshall T. H. (2021). Citizenship and Social Class, and Other Essays. Melbourne, VIC: Hassell Street Press.

[B32] Mead G. H. (1934). Mind, Self, and Society: From the Standpoint of a Social Behaviorist. Chicago, IL: University of Chicago Press.

[B33] Ministry of Education Culture, Sports, Science and Technology. (2024). Survey on the Acceptance of Children and Students Requiring Japanese Language Instruction. Available online at: https://www.mext.go.jp/b_menu/houdou/31/09/1421569_00006.htm (Accessed August 13, 2025).

[B34] Ministry of Health Labour and Welfare. (2024). Statistics on foreign workers in Japan. Ministry of Health, Labour and Welfare. Available online at: https://www.mhlw.go.jp/stf/newpage_50256.html (Accessed August 29, 2025).

[B35] Ministry of Internal Affairs and Communications (2020). Revised Plan for the Promotion of Multicultural Coexistence in Local Communities. Available online at: https://www.soumu.go.jp/menu_seisaku/chiho/02gyosei05_03000060.html (Accessed August 13, 2025).

[B36] Ministry of Internal Affairs and Communications (2022). Tabunka kyō*sei suishin ni kansuru ch*ō*sa h*ō*kokusho [Survey Report on the Promotion of Multicultural Coexistence]*. Ministry of Internal Affairs and Communications. Available online at: https://www.soumu.go.jp/main_content/000718726.pdf (Accessed August 29, 2025).

[B37] Ministry of Internal Affairs and Communications (2024). Tabunka kyō*sei suishin ch*ō*sa [Survey on Multicultural Coexistence Promotion]*. Ministry of Internal Affairs and Communications. Available online at: https://www.soumu.go.jp/menu_seisaku/chiho/02gyosei05_03000060.html (Accessed August 29, 2025).

[B38] Ministry of Internal Affairs and Communications (MIC) (2006). Promotion of Multicultural Coexistence in Local Communities. Available online at: https://www.soumu.go.jp/ (Accessed August 13, 2025).

[B39] Ministry of Justice (2024). Statistics on Foreign Residents in Japan. Available online at: https://www.moj.go.jp/isa/ (Accessed August 13, 2025).

[B40] MIPEX (2020). Migrant Integration Policy Index 2020. Migration Policy Group. Available online at: https://www.mipex.eu/ (Accessed August 29, 2025).

[B41] Miyajima T. (2004). Zainichi gaikokujin no h$\bar{\text{o}}$*teki chii to shakai teki t*$\bar{\text{o}}$*g*$\bar{\text{o}}$ *[The Legal Status and Social Integration of Foreign Residents in Japan]*. Tokyo: Tokyo Daigaku Shuppankai.

[B42] Miyajima T. (2009). Tabunka ky$\bar{\text{o}}$sei no kadai: Nihon to $\bar{\text{O}}$sh$\bar{\text{u}}$ o megutte [The problems and challenges of multicultural coexistence: a view from Japan and Western Europe]. Kagaku 14, 12–16. Available online at: https://www.jstage.jst.go.jp/article/tits/14/12/14_12_12_10/_pdf (Accessed August 13, 2025).

[B43] Modood T. (2007). Multiculturalism: A Civic Idea. Cambridge: Polity Press.

[B44] Multicultural Center Tokyo (2025). Official Website. Available online at: https://tabunka.or.jp/ (Accessed August 13, 2025).

[B45] OECD (2018). Working Together for Local Integration of Migrants and Refugees. OECDPublishing. doi: 10.1787/9789264085350-en38483583

[B46] OECD (2021). International Migration Outlook 2021. OECDPublishing. Available online at: https://www.oecd.org/en/publications/international-migration-outlook-2021_29f23e9d-en.html (Accessed August 29, 2025).

[B47] OECD (2024). International Migration Outlook 2024. OECDPublishing. Available online at: https://www.oecd.org/en/publications/international-migration-outlook-2024_50b0353e-en.html (Accessed August 29, 2025).

[B48] Oguma E. (2002). A Genealogy of “Japanese” Self-Images. Tokyo: Trans Pacific Press.

[B49] Parekh B. (2000). Rethinking Multiculturalism: Cultural Diversity and Political Theory. Cambridge, MA: Harvard University Press.

[B50] Raz J. (1986). The Morality of Freedom. Oxford: Clarendon Press.

[B51] Ringstrom A. Johnson S. (2022). Swedish Moderates Make Coalition Deal, Anti-immigration Party to Back New Govt. Available online at: https://www.reuters.com/world/europe/swedish-moderates-party-leader-strikes-deal-form-minority-government-2022-10-14/ (Accessed August 13, 2025).

[B52] Saga Prefecture International Relations Association (SPIRA) (2025). Official Website. Available online at: https://www.spira.or.jp/ (Accessed August 13, 2025).

[B53] Siddle R. M. (2014). Race, Resistance and the Ainu of Japan. London: Routledge.

[B54] Smith A. (2002). The Theory of Moral Sentiments. Cambridge: Cambridge University Press. (Original work published 1759).

[B55] Statistics Bureau of Japan (2024). Population Estimates as of January 2024. Available online at: https://www.stat.go.jp/data/jinsui/ (Accessed August 13, 2025).

[B56] Takaya Y. Higuchi N. Inaba N. Okunuki A. (eds.). (2019). Imin seisaku to wa nanika: Nihon no genjitsu kara kangaeru [What Is Immigration Policy? Reflections From the Japanese Context]. Tokyo: Jinbun Shoin.

[B57] Taylor C. (1994). Multiculturalism: Examining the Politics of Recognition. Princeton, NJ: Princeton University Press.

[B58] Thaler R. H. Sunstein C. R. (2008). Nudge: Improving Decisions About Health, Wealth, and Happiness. New Haven, CT: Yale University Press.

[B59] The Guardian (2010). Angela Merkel: *German Multiculturalism Has ‘Utterly Failed'*. Available online at: https://www.theguardian.com/world/2010/oct/17/angela-merkel-german-multiculturalism-failed (Accessed August 13, 2025).

[B60] Tocqueville A. D. (2000). Democracy in America. Chicago, IL: University of Chicago Press. (Original work published 1835).

[B61] Town of Ōizumi (2023). Overview of the Town. Available online at: https://www.town.oizumi.gunma.jp/ (Accessed August 13, 2025).

[B62] Tsuda T. (2003). Strangers in the Ethnic Homeland: Japanese Brazilian Return Migration in Transnational Perspective. New York, NY: Columbia University Press.

[B63] Vertovec S. Wessendorf S. (eds.). (2010). The Multiculturalism Backlash. London: Routledge.

[B64] Yokohama City (1991). Fundamental Education Policy for Foreign Residents in Yokohama City. Yokohama CityBoard of Education. Available online at: https://shinaijuku.com/common/img/document/190604_Shinaijuku_40th_Hoshin.pdf

[B65] Yomiuri Shimbun (2023). Toruko-jin gurū*pu de toraburu, by*ō*in ni 100-nin ga atsumari sawagi ni... satsujin misui yogi no dansei ra 7-nin fukiso [Trouble Among Turkish Group: About 100 People Gathered at Hospital After Incident–Seven Men, Including Suspect in Attempted Murder Case, Not Indicted]*. The Yomiuri Shimbun. Available online at: https://www.yomiuri.co.jp/national/20230926-OYT1T50208/ (Accessed August 29, 2025).

